# Recent Progress of Biomarker Detection Sensors

**DOI:** 10.34133/2020/7949037

**Published:** 2020-10-15

**Authors:** Ruitao Liu, Xiongying Ye, Tianhong Cui

**Affiliations:** ^1^State Key Lab Precise Measurement Technology & Instrument, Department of Precision Instruments, Tsinghua University, Beijing, China; ^2^Department of Mechanical Engineering, University of Minnesota, Minneapolis, Minnesota, USA

## Abstract

Early cancer diagnosis and treatment are crucial research fields of human health. One method that has proven efficient is biomarker detection which can provide real-time and accurate biological information for early diagnosis. This review presents several biomarker sensors based on electrochemistry, surface plasmon resonance (SPR), nanowires, other nanostructures, and, most recently, metamaterials which have also shown their mechanisms and prospects in application in recent years. Compared with previous reviews, electrochemistry-based biomarker sensors have been classified into three strategies according to their optimizing methods in this review. This makes it more convenient for researchers to find a specific fabrication method to improve the performance of their sensors. Besides that, as microfabrication technologies have improved and novel materials are explored, some novel biomarker sensors—such as nanowire-based and metamaterial-based biomarker sensors—have also been investigated and summarized in this review, which can exhibit ultrahigh resolution, sensitivity, and limit of detection (LoD) in a more complex detection environment. The purpose of this review is to understand the present by reviewing the past. Researchers can break through bottlenecks of existing biomarker sensors by reviewing previous works and finally meet the various complex detection needs for the early diagnosis of human cancer.

## 1. Introduction

The health of human beings is always being one of the more complicated topics in modern science. Important bio-information which is represented by DNA, cells, or biomarkers has become the focus of bioscience research. In most bioresearch fields, especially for cancer detection, lots of biological molecular (like DNA and biomarkers) and other physiological markers have been proven useful for cancer diagnosis and management [1–4]. Among these molecular analytes, biomarkers are often considered as a kind of quantifiable label that indicates certain biological states of human bodies. Therefore, biomarker sensors hold enormous potential for early diagnosis and personalized therapy to disease [5–7]. Biomarkers not only offer us information about existing diseases, more importantly, they provide individualized information regarding underlying medical conditions. By analyzing results between normal samples and patients, this information will provide morbidity, sub-clinical status, and other biology information to users in a rapid fashion. Therefore, the detection of biomarkers is of great significance to human health.

**Figure 1:**
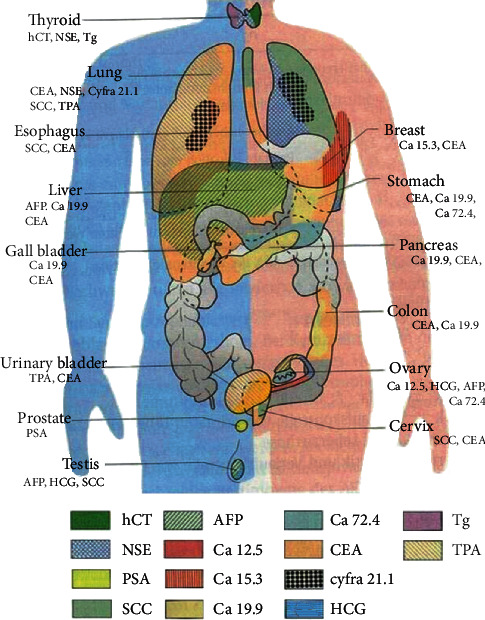
Various biomarkers in the human body. Reference from tumor immune cell therapy website.

As shown in Figure 1, biomarkers are widely distributed in various organs of human bodies. In the medical diagnostics field, the concentration of one kind of biomarker is not enough to confirm cancer; thus, it is necessary to detect multibiomarkers simultaneously for early diagnosis. In the real biological environment, the concentration of these biomarkers is always limited to a narrow range in a healthy individual. Some detailed information is shown in Table 1.

Regarding the reference ranges which are shown in Table 1, there are two types of biosensor-based applications in the modern medical diagnosis field: one is an extremely accurate and precise detection biosensor and another is a low-cost, real-time and in-site sensing device [14]. The former biomarker sensors are beneficial for us to investigate the mechanism of diseases and are the gold standard for the status of the biomarker itself, while the latter ones meet the needs of early diagnosis or community screening. Nowadays, some commercial biomarker sensors based on electrochemistry, surface plasmon resonance (SPR), nanowires, and other microstructures have already been used in biological information detection and medical fields. For example, SPR biosensors are used in ultrahigh precise detection areas due to their ultrahigh sensitivity and ultralow limit of detection (LoD). And electrochemistry-based biosensors are also used in cancer screening with low-cost, rapid response and high throughput.

**Figure 2:**
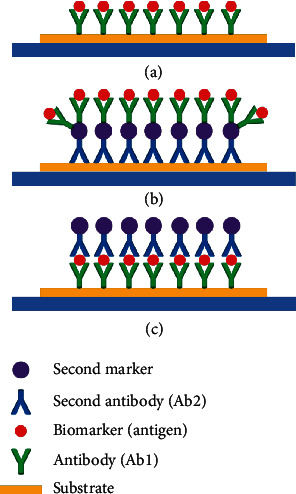
Schematic diagram of label-free (a) and label-based biosensors. (b, c) show different strategies of secondary label decoration.

As one of the most effective methods to obtain biological information, biomarker detection has been investigated and developed for many years. In recent years, combining modern materials or secondary label enhancement technologies, lots of traditional biomarker sensors based on SPR and electrochemistry optimize their performance in sensitivity, LoD, and linear range. Although these biomarker sensors work using different principles, they still can be classified into two categories: label-based and label-free sensors which are shown in Figure 2.

Label-based sensors use biomarkers that are decorated by secondary labels, such as fluorescent materials, magnetic beads, or even biological materials. These labels optimize the sensitivity by amplifying the output signals, such as the 1000-fold increase in sensitivity when detecting the decorated prostate-specific antigen (PSA) [15]. Through the development of 2D materials and microfabrication technologies, some new secondary labels exhibit a great deal of potential in biomarker detection due to their better biocompatibility, larger surface-to-volume ratio, etc. [16]. These secondary labels have been widely used in laboratories to optimize sensitivity, limits of detection, and dynamic range. Secondary labels include carbon nanotube, graphene oxide, nanoparticles (NPs), and quantum dots (QDs) [17]. Combining these novel labels, there are two core questions that need to be answered: how do we improve the capture efficiency of biomarkers and how do we enhance output signal intensity for the unit biomarker. However, we cannot ignore the background noise, nonspecific adsorption, and environment interference that come with these new labels.

Compared to the labelled biomarker sensors, label-free biomarker sensors can be used in more complex detection environments [18–21]. In a label-free detection system, the concentration of biomarkers is transferred into the changes of the properties of the material itself or changes in the microstructure of these sensors. By measuring these changes, different outputs will be achieved which may have a linear or nonlinear relationship with the concentration of biomarkers under certain excitation conditions. Similarly, to label-based sensors, optimization of capture efficiency is also a key point in label-free sensor research. Furthermore, it is possible to fabricate more complex three-dimensional micro- or nanostructures based on the development of microfabrication. Hence, some novel label-free micro- or nanostructure-based biomarker sensors—such as cantilever biosensors, nanowire-based field-effect transistor sensors, and metamaterial biosensors—offer us new ideas.

In this review, some biomarker sensor research has been reviewed and classified by their mechanism of detection. Combining optimized electrode modification and biomarker decoration technologies, electrochemical biomarker sensors have made considerable progress in detection sensitivity and LoD. Besides, microfabrication technologies not only enrich our method for biomarker sensor design but also provide many innovative microstructures as well, for example, microfluid- and nanofluid-based biosensors, which optimize stability and reproducibility when they are assembled into commercial sensors. All in all, as the development of modern materials and microfabrication technologies advance, biomarker sensors and platforms will not only break through current challenges but also exhibit tremendous potential for the early diagnosis of cancer and individualized health monitoring as well.

## 2. Electrochemical Biomarker Sensors

### 2.1. Basics of Electrochemical Biomarker Detection

Specific binding is the principle behind various biomarker detection techniques which allow us to capture specific targets from a natural selection. One of the most widely used biomarker detection technologies, electrochemistry-based biomarker sensors are used in a variety of detection areas, including food safety [22], environment protection [23], and even space exploration [24]. Based on several detection methods, electrochemistry-based biomarker sensors can be classified as voltammetry-linear sweep, differential pulse, square wave, stripping and amperometry, and so on. Regardless of their classification, most of these label-based sensors work as follows: (1) the secondary antibody (Ab2) is decorated with electroactive labels, such as biochemical materials and nanoparticles of novel 2D materials; (2) the Ab2 is bound on the analyte through an intermediate Ab1 and then is immobilized onto the electrode surface by specific binding; and (3) the concentration of the analyte is obtained by measuring the current at the electrode [25].

In recent years, some electrochemistry-based biomarker sensors have been designed and fabricated, which could be classified into two categories based on the optimizing strategy: (1) Increasing the capture efficiency on the surface of devices. Ideally, a biomarker sensor can only capture biomarkers by specifically binding and does not capture any other impurities. However, there are always inevitable factors like impurities in the buffer solution, vibrations, and changes of temperature and humanity and so on from the detection environment. Among these factors, although researchers try their best to build a barrier layer (such as bovine serum albumin (BSA) or other materials) to reduce interference, nonspecific binding is still a concern. (2) Increasing the intensity of the output signal under the same concentration of biomarkers. Fluorescent labels have the same function as an amplifier; the more fluorescent labels that are detected by charge-coupled devices (CCD), the larger the electric signals that will be generated, so researchers try to increase the number of fluorescent labels on the surface of the second antibodies (as is shown in Figure 2(c)) to generate a larger output signal at the same concentration of biomarkers and then optimize the resolution and limit of detection. Based on two strategies which are mentioned above, some novel electrochemical biomarker sensors have been fabricated as follows.

### 2.2. Electrode Modification Strategy for Biosensors

Some researchers optimize the substrate or electrodes by pretreatment to reduce interference or background noise. No matter which analytes are prepared in the laboratory, serum or urine, there are still some nonbiomarkers or impurities in the buffer that attach onto the surface of the substrate or electrodes. This will enlarge background noise and then shorten the LoD of sensors or even show error information when the noise is the same level as effective signals. Therefore, some researchers tried to optimize the substrate material through the biological- or chemical-based pretreatment which has proven to be an effective strategy to reduce interference from the background.

**Figure 3:**
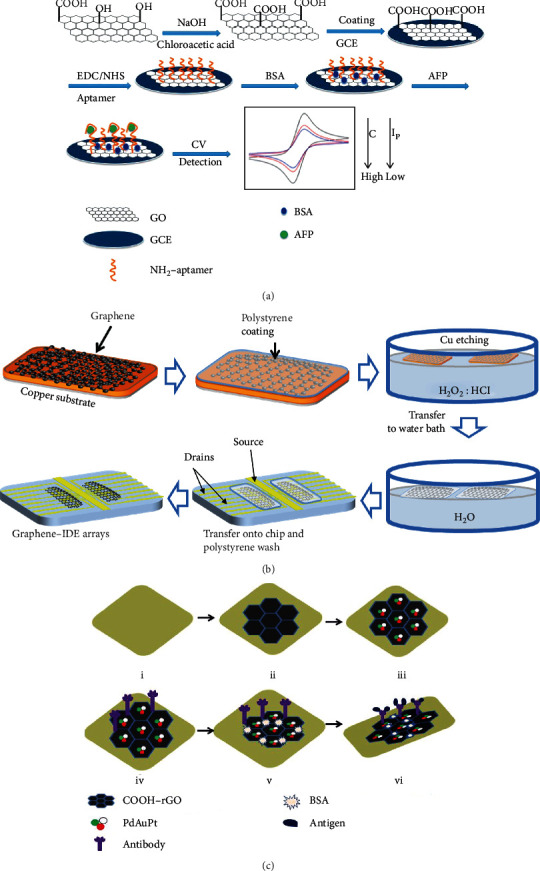
Electrode modification strategies with different types of materials. (a) 2D material GO and aptamer modified on the GCE, reproduced from Yang et al. [26]. (b) Graphene-based 2D material modified chip by wetting transfer, reproduced from Delle et al. [27], and (c) rGO and nanocomposites Pd, Au, and Pt immobilized on the electrode, reproduced from Barman et al. [28] with their permissions.

Due to better electron transport property and biocompatibility of some 2D materials, some novel biosensors with 2D materials had been fabricated. Klukova et al. predicted that the graphene could enrich the fabrication of the lectin-based biosensor, because it can provide many different methods for lectin immobilization [29]. As shown in Figure 3(a), Yang et al. developed a noise-suppressing AFP sensor with a large binding area by decorating its substrate with graphene oxide (GO) [26]. Wu et al. used a synthesized Au/AgrGO as electrode material to load graphene quantum dots and then amplified the ECL signal [30]. Vasantham's group developed a paper-based sensing platform for cTnI detection by using *μ*PAD and multiwalled carbon nanotubes (MWCNT) [31]. In their work, the substrate is made of paper which improves economic viability and also makes for an extremely sensitive approach to facilitate clinical decision making. Due to its good biocompatibility, high conductivity, and large area of effective surface, single-layer reduced graphene oxide (rGO) was modified on a glassy carbon electrode to develop a Tau-441 sensor by Ye et al. In addition, Cu ion also enhanced its performance in their work [32]. Wang and coworkers developed an origami-paper-based electrochemical biomarker sensor by modifying the electrode with NH2-GO/thionine/GNP. By this method, the paper substrate could generate output signals and offer the active sites for EGFR aptamer immobilization [33].

Besides 2D materials, some researchers with biological background have brought biochemical materials into biomarker detection which have exhibited tremendous potential in the field of clinical medicine. Shoja et al. fabricated a DNA biosensor with high electrical conductivity and large area of an effective surface based on rGO/functionalized mesoporous carbon/Ni-oxytetracycline metallopolymer nanoparticle-modified pencil graphite, immobilized onto the surface of electrodes [34]. Combined with chemical vapor deposition (CVD), graphene has been synthesized on Cu film and immobilized anti-CEA which linked PBSE. Singh et al. fabricated an anti-CEA/PBSE/graphene/Cu biomarker detection platform for CEA detection based on electrochemical impedance spectroscopy (EIS) technique [35]. Delle et al. transferred multilayer graphene, as a high surface-to-volume ratio electrical layer, onto interdigitated electrode arrays to improve electrical conductivity, which is shown in Figure 3(b) [27]. Aydın et al. decorated the ITO electrode with composite material (carbon black and PGMA polymer) for anti-IL-8 antibody immobilization and then fabricated a high-sensitivity label-free biosensor for IL-8 detection [36]. Taking advantage of the excellent specificity with negligible interference from the serum of Affimer proteins, Zhurauski et al. developed an Affimer-based electrochemical biosensor for Her4 detection combined with non-Faradaic impedance and ID*μ*Es [37]. Seenivasan et al. developed a prostate-specific membrane antigen (PSMA) sensor, in which gold nanoparticles (GNPs) functionalized with PSMA antibodies (PSMA-Ab) were decorated onto patterned electrodes (ITO-coated glass) to improve the limit of detection [38]. Aydın fabricated an electrochemical sensor for interleukin 1*α* (IL-1*α*) detection. In their work, the indium tin oxide electrode was modified by epoxy-substituted polythiophene polymer which shows good immobilization because of the epoxy group on the side of this polymer [39]. Jolly et al. fabricated a synthetic receptor sensor with a threefold increment of sensitivity for aptamer-PSA complex capturing by controlling electropolymerization. In their work, biological molecular recognition and molecular imprinting technologies had also been used [40]. Combining various nanoparticles (NPs) or nanocomposites, the electrochemical biomarker sensors, especially those ECL- and PEC-based sensors, have better performance in limits of detection (LoD) and a linear range. Some electrochemical studies also show that these composites have “excellent electrochemical properties such as a large electrochemically active surface, high capacitance current, a wide potential window, high conductivity and large porosity” [41]. Moreover, by combining with some 2D materials, catalysis or positron-electron recombination management is generated which further optimizes the performance of these sensors. Tran et al. fabricated a CEA sensor based on Cu-Au/CNT-CS hybrid material. In their work, CNT-linked CSs improved electroactive surface area and electrical conductivity. As a catalyst, Cu not only impacts the growth density of CNT but also provides conductive pathways to accelerate electron transfer between the redox probe and the surface of the electrode [42]. Taking advantage of more electroactive sites and a larger surface area for biomarker captured, Barman et al. developed a CEA and PSA biosensor by using Pd, Au, and Pt nanocomposites which were immobilized onto the electrodes decorated by rGO, which is shown in Figure 3(c) [28]. Wu et al. developed a label-free photoelectrochemical sensor for CEA detection, which was fabricated by using 2D graphitic carbon nitride nanosheets (g-C_3_N_4_) with Zn_0.1_Cd_0.9_S nanocrystals. Due to the excellent photoelectron-chemical activity of as-synthesized Zn_0.1_Cd_0.9_S/g-C_3_N_4_ composites, the separation and transfer of photogenerated electron-hole pairs had been accelerated [43]. Feng et al. proposed an aminoterminal probrain natriuretic peptide sensor based on La-CdS/3D ZnIn_2_S_4_/Au@ZnO. In their work, charge recombination had been depressed because of excellent photoelectric activity, the electrical conductivity of Au@ZnO, and the large surface for deposition of La-CdS [44]. Liu et al. developed a label-free PEC sensor for nucleoside diphosphatase kinase A (NDPK-A) detection. In their work, ZnO nanorod arrays, as electrodes with a large surface area of the nanostructure, demonstrated a high and stable photocurrent response. And combined with CdS nanoparticles, they further improved photocurrent response based on the perfect matching of energy levels between ZnO and CdS [45]. Hu et al. developed a PEC sensor for HBsAg detection by using ZnAgInS QDs—a photoactive material. In their work, gold nanoparticles played a role as a catalyzer to enhance the photocurrent response and also enlarged active sites for anti-HBsAg immobilizing [46]. By assembling Ag_2_S nanoparticles with titanium dioxide and bismuth vanadate (TiO_2_/Ag_2_S-BiVO_4_) on the electrode, Feng et al. developed a label-free PEC sensor for ochratoxin-A quantitative detection. In their work, ascorbic acid acted as the electron donor for inhibiting holes' generation and recombination of electron-hole pairs [47]. In the work of Patra et al., the multiwall carbon nanotubes (MWCNTs) with manganese nanoparticles were decorated with thio groups to make a nanoiniferter, which was used to synthesize a three-dimensional molecularly imprinted polymer (MIP) matrix for trace level detection of PSA [48]. To accelerate the electron transfer, Wu et al. modified MWCNTs, polyamidoamine dendrimer, and golden nanoparticles on a glassy carbon electrode (GCE). This modified electrode not only provided enough amine groups for biomarker immobilization but also showed the high recovery efficiency because of the peptides labeled with golden nanorods (acted as an acceptor) [49]. Taking advantage of the synergetic effect which was presented from the Cu_2_O@CeO_2_ core-shell decorated with GNPs (Cu_2_O@CeO_2_-Au), Li et al. showed a better electrocatalytic activity to reduce hydrogen peroxide (H_2_O_2_) than single Cu_2_O, GNPs, or Cu_2_O@CeO_2_ [50]. Govindasamy et al. fabricated a progesterone sensor with cyclic voltammetry and amperometric measurement; electrodes of this sensor were modified by WO_3_ NBs@GR nanocomposite electrocatalyst [51]. And in the same year, Govindasamy et al. developed another electrochemical sensor for *α*-amino acid detection by using SrTiO_3_@rGO-modified electrode. In their work, these nanocomposites played a vital role in the improvement of sensor performance [41]. Sriram et al. exhibited wonderful performance with biomarkers of uric acid, using the Fe_3_O_4_ NPs@rGOs, as an electrocatalyst which was modified onto GCE and amplified output signals [52]. With the same idea, Mani et al. successfully developed an excellent electrocatalyst-based biosensor for dopamine detection [53]. Govindasamy et al. fabricated nonenzymatic biosensors based on ZnO and nanoflower@GO nanocomposites for the determination of cancer markers [54]. Employing enhanced electron transport and interfacial properties of a MoS_2_ interface with electrodeposited Au nanoparticles, Yagati et al. fabricated triiodothyronine detection bioassays with Au-MoS_2_-modified ITO electrodes by electrodeposition procedures [55]. Taking advantage of the hierarchical nanohybrid structure and dual nanoenzyme activities, Zhao et al. fabricated a graphene fiber electrochemical sensor, modified with MnO_2_-NWs@Au-NPs, for H_2_O_2_ detection. The flower-like MnO_2_-NWs showed superior catalytic activity and offered a large surface area for Au-NP decoration [56].

In addition to some labels described above, electrochemical biomarker sensors can also optimize their performance by combining with modern microfabrication techniques [57–60]. Kuo et al. focused on electrode improvement with interdigitate-zigzag and developed a high sensitivity electrochemical biosensor for CVD biomarker detection [61]. Arya et al. fabricated a PMMA array sensor for tumor necrosis factor-alpha (TNF-*α*) detection, which effectively reduces most background noise due to the specific binding of the substrate [62]. By using a 3D carbon system, a new biosensor strategy for signal amplification was developed by Sharma et al. for cMyo detection, which consisted of a suspended mesh and interdigitated array (IDA) nanoelectrodes [63]. Shi et al. developed a highly sensitive electrochemiluminescence (ECL) biosensor based on a closed bipolar electrode structure for PSA detection. The bipolar electrode was modified to produce two different stages of enhancement on the same electrode [64]. There are other interesting research being done, like Liu and Lillehoj who came up with a novel method to design an electrochemical based biosensor, which could be used as a wearable application from its excellent flexibility and durability [65]. More than that, combining with high flexibility of polymer and good biocompatibility of the metal, Tang et al. successfully developed a metal-polymer conductor for biosensing application. In their work, this printed conductor inhibited the surface tension of liquid metal and also avoided the particles' sintering [66]. Besides this, molecular imprinting (MIP) is a method of preparing polymer materials containing predetermined selective recognition sites. This method functionalizes the surrounding of the template monomer and fixes and assembles with the polymer material by self-assembly. The monomer is then extracted from the polymer, leaving a recognition site that can rebind the analyte [67]. Reducing the loss of activity of the biological analyte and the long-term stability of the recognition layer are the advantages of this technology [68]. Based on MIP technology, some biosensors with excellent performances also had been fabricated [69–74].

### 2.3. Biomarker Decoration Strategy of Sensors

Besides modifying the electrodes or substrate, another strategy is secondary antibody (Ab2) decoration. Compared to traditional electrochemical biomarker sensors, decorating Ab2 is an effective way to optimize the sensitivity and limit of detection. Some researchers have shown that the biomarker decoration strategy can offer new opportunities for highly sensitive protein detection by combining with biochemical labels, 2D materials, nanoparticles, etc. [75–78]. In recent years, many electrochemical biomarker sensors have been developed with different secondary labels, such as biomaterials, two-dimensional materials, and fluorescence materials. For example, the magnetic bead is usually used on substrates for antibody capture or target antigen [79–83] and then shows a faster reaction, greater surface area per unit volume, and better stability when compared to bulk solid surfaces [84, 85]. Moreover, the magnetic bead is more easily modified with functional groups, like DNA, enzymes, or antibodies, which makes a great substrate for the development of sensitive, rapid electrochemical biosensors [86, 87]. Multienzyme, as an extra label, is immobilized onto the surface of the materials or microstructure, which could optimize the limit of detection and sensitivity [88, 89]. More than that, secondary labels also make great contributions to electrochemiluminescence (ECL) biosensors and photoelectrochemistry biosensors (PEC). In recent years, lots of applications based on ECL and PEC have been significantly developed, and several excellent reviews have already reported on the mechanism, advantages, and applications of this highly sensitive and selective detection method [90, 91]. And these improved electrochemistry-based biomarker sensors change the detection mechanism of original signals, which could both enrich their design and improve performance.

**Figure 4:**
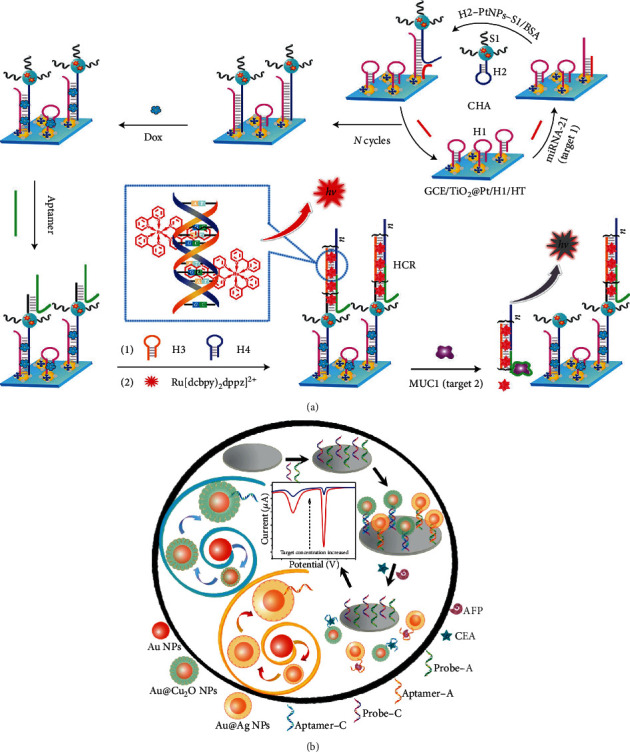
Secondary label decoration strategies with different types of materials. (a) ECL-based biomarker sensor amplified its output by decorating biology material and NPs on the biomarker, reproduced from Nie et al. [92]. (b) Combing with the GNPs, the biosensor was optimized by controlling the thickness of the shell, reproduced from Zhao et al. [93] with their permissions.

As is shown in Figure 4(a), Nie et al. successfully designed a versatile and ultrasensitive ECL sensing platform to monitor breast cancer biomarkers by using a nonenzymatic catalytic hairpin assembly and hybridization chain reaction for amplifying [92]. Combing with nanoparticles, biomaterial-based biosensors also exhibit potential for progress. As shown in Figure 4(b), by controlling the thickness of the shell upon the GNPs, Zhao et al. optimized the detection sensitivity for both CEA and AFP in their sensor [93]. Chuang et al. fabricated a TNF-*α* sensor by measuring the diffusivity, in which is an immobilized biomarker with and without GNPs on the surface of particles [94]. Tan et al. fabricated a cTn1 sensor based on PEC; a newly developed photocurrent-enhancing nanocomposite was used as a PEC transducer which was made from N-acetyl-L-cysteine capped CdAgTe QDs and dodecahedral GNPs in the sensor. In addition to these novel nanoparticles, biomarker sensors can also be optimized by improving Ab2 decorated with other materials, such as polymers [95]. Zou et al. proposed a new signal enhancement strategy for protein biomarkers assay binding based on a metal-ion-dependent DNAzyme recycling [96].

Combining QDs, NPs, and other micro- or nanoparticles, some novel two-dimensional materials greatly increase specific surface area and effective detection area, thereby increasing output signals of electrochemistry or photoelectrochemistry.

**Figure 5:**
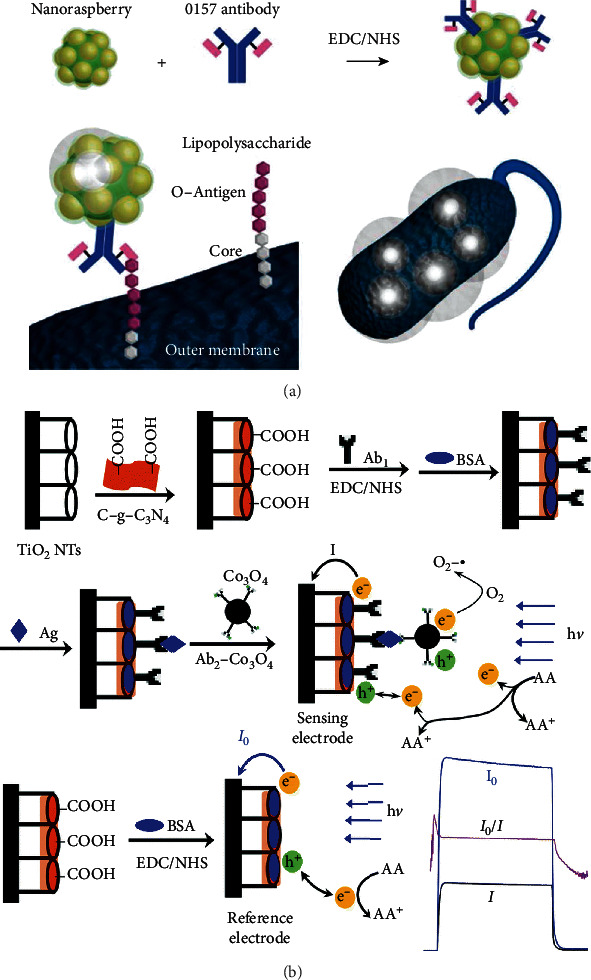
Light intensity detection and PEC-based sensors. (a) is a nanoantenna through antigen-antibody reaction on a bacterial surface, reproduced from Shiigi et al. [97], and (b) is a PEC-based biomarker sensor, reproduced with permissions from Wu et al. [98]; the NPs could assist the consumption of electron donors.

As shown in Figure 5(a), Shiigi et al. demonstrated a raspberry-shaped nanostructure with a high density of GNPs acting as an excellent antenna. Due to the aggregation and dispersion states of this raspberry-shaped nanostructure, the characteristic optical properties could provide useful information regarding bacteria and permit sensitive detection for bacterial cell analysis [97].  Figure 5(b) shows that Wu et al. fabricated a PEC sensor for AFP detection coupled with secondary antibody-Co_3_O_4_ nanoparticle conjugates (Ab2-Co_3_O_4_ NPs) due to their steric hindrance effect and the consumption of electron donors for signal amplification [98]. Sharafeldin demonstrated an electrochemistry-based biosensor by using magnetic Fe_3_O_4_/GO composites as the secondary label. In their work, Fe_3_O_4_ nanoparticles provided precise control for the number per GO sheet and optimized the dynamic range of the sensor [99]. Kooshki et al. decorated the second label with nanobiomaterial-silica NP/graphene oxide to improve sensitivity 10^5^ times due to the increment of active surface, facilitation of electron transference rate, and high biocompatibility [100]. As nanoparticles can generally amplify the signal in an electrochemical sensor, Freitas et al. fabricated a time-saving (a total time assay of 2 h) electrochemical biomarker sensor for HER2 detection by decorating Ab2 with core/shell CdSe@ZnS quantum dots as the electroactive label [101].

### 2.4. Double Strategies of Sensors

In order to further improve the performance of biomarker sensors, dual optimization strategies have been developed by most researchers. Among the dual strategy electrochemical biomarker sensors, two-dimensional materials and composite materials are widely used, such as carbon nanotube, golden nanoparticles, and reduced graphene oxide.

**Figure 6:**
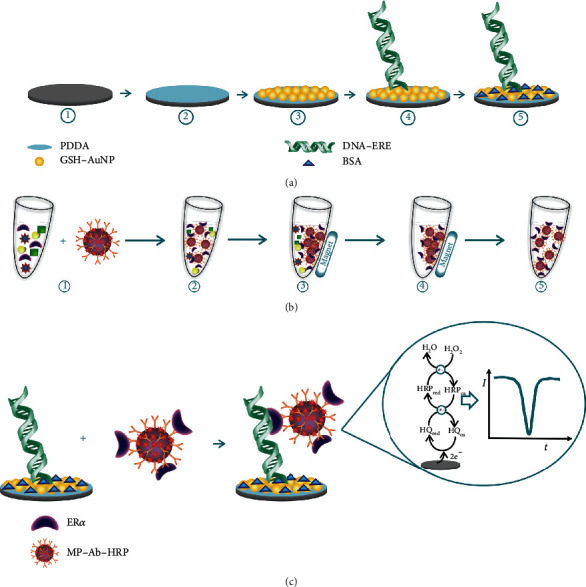
Dual amplification strategy. (a) Immobilization of substrate or electrodes by PDDA and GNPs. (b) Decoration of secondary labels-magnetic particles-Ab1-HRP and (c) combination of the above two parts and detection of ER*α* with different concentrations, reproduced from Uliana et al. [102] with permission.

As is shown in Figure 6, Uliana et al. developed a disposable microfluidic sensor for ER*α* detection. In their work, HRP and magnetic particles decorated with a primary antibody (Ab1) were used to capture analytes and amplify signals, and the ER*α* was recognized based on the DNA-ER*α* interaction on the surface of electrodes [102]. In the work of Jiang et al., a novel electrochemical AFP sensor was developed with an ultrasensitive sandwich structure, in which the substrate was modified by b-cyclodextrin functionalized graphene and platinum hybrid MWCNTs adhered to copper oxide (Pt@CuO-MWCNTs) as the secondary label. Due to the catalyst of Pt, CuO, and MWCNTs for the decomposition of H_2_O_2_, the Ab2 made multiple amplifications for signals [103]. Taking advantage of the better coverage of carbon nanomaterial (GNP included) and the larger accessible surface area for antibody immobilization, Freitas et al. fabricated an electrochemical-based HER2 sensing platform on screen-printed carbon electrodes with Ab2-MWCNT/GNP for breast cancer detection [104]. Sardesai et al. immobilized biomarkers onto the surface of CNTs which was modified with fluorescent labels and then used to detect IL-6 and PSA by measuring the ECL intensity [105]. Zhu et al. fabricated an electrochemical biosensor by using the Hyd-GNP-Apt bioconjugate for breast cancer biomarker detection—HER2 and SK-BR-3. The hydrazine reductant was decorated onto GNPs to inhibit nonspecific binding of silver on the surface of the electrode, which selectively amplify signals [106]. Chen et al. connected a specific aptamer sgc8c with the rGO-dendrimer interface to improve the capture efficiency and enhance the electron transfer ability. Besides that, lectins and alkaline phosphatase-modified gold nanoparticles were also prepared as signal probes that inhibit the Ru(bpy)_3_^2+^ ECL reaction in those experiments [107]. Fang et al. fabricated a biosensor platform with Au/ZnO/rGO acting as substrate and Ab2/HRP-Au@ZnO working as the secondary label. In their work, Au/ZnO/rGO nanocomposite increased the loading capacities of Ab1 and Au@ZnO carrier increased the amount of captured Ab2/HRP bioconjugate, which also possessed peroxidase-like catalytic activity to enhance the output signals [108]. Lv et al. fabricated a CEA sensor based on the dendritic Au@Pt functionalized nitrogen-doped graphene loaded with copper ion (Au@Pt DNs/NG/Cu^2+^) which acted as the secondary label to enhance peroxidase-like properties. And the Au@polydopamine decorated substrate also exhibited good conductivity and adhesion to Ab2 in a large surface area, which greatly amplified the electrochemical signal [109]. Zhang et al. designed an ultrasensitive sandwich electrochemical sensor by using a decorated substrate with GNPs which can effectively immobilize antibodies and accelerate electron transfer. Moreover, Au@Ag nanoparticles loaded by polydopamine functionalized phenolic resin and microporous carbon spheres (Au@Ag/PDA-PR-MCS) were also used as the secondary labels to amplify the current signal [110]. Sun et al. fabricated a highly sensitive PEC sensor for avian virus detection based on dual strategies. In their work, ALP-CdTe-Ab2 was used not only to enhance PEC signals but also to produce electron donors due to the enzymatically catalytic characteristic. Besides that, GNPs/g-C_3_N_4_ hybrids also acted as a basic photoactive layer to improve absorption photocurrent response [111]. In the work of Fan et al., a PEC composite with ultrasensitive photoelectrical response was fabricated by using carboxyl functionalized CdSe QDs enhanced La-TiO_2_, and carboxyl modified polystyrene microspheres were coated by Ab2 for PSA detection [112]. Feng et al. modified Au/thionine functionalized graphene oxide on the substrate to accelerate the electron transfer and decorated PtCu/reduced graphene oxide/graphitic carbon nitride (PtCu@rGO/g-C_3_N_4_) on Ab2 was used to amplify signals due to its ability to reduce H_2_O_2_ [113]. By coating graphene oxide/ssDNA on the golden electrode and incorporating with poly-L-lactide nanoparticles, Pan et al. amplified the signal for vascular endothelial growth factor (VEGF) and PSA detection [114]. Zhou et al. amplified the signal of their sensor based on the rGO-BiFeO_3_-acted as the photoactive material- and target-triggered hybridization chain reaction (HCR). From their explanation, rGO had a great contribution to accelerate the charge transfer and enhanced absorption under visible light irradiation [115]. By decorating the rGO doped with Ca ions, Wang et al. successfully both improved the conductivity and inhibited the recombination of electron-hole pairs of CdSe nanoparticles, thereby enhancing the photocurrent conversion efficiency [116].

Over the past decade, with the development of microfabrication and investigation of modern materials, electrochemistry-based biomarker sensors have made great progress.  Table 2 shows LoD and linear range of electrochemistry-based biosensors for PSA, AFP, and CEA detection. Although the complex pretreatment of labels and modification of electrodes limits the electrochemical biomarker sensor to some extent, the electrochemical mechanism-based sensors are still widely used in a variety of biomarker detection fields as a low-cost, reliable, portable, multiplexed protein detection sensor.

## 3. Enzyme-Linked Immunosorbent Assay-Based Biomarker Sensors

Enzyme-linked immunosorbent assay (ELISA), a kind of commonly used analytical biochemistry assay, takes advantage of the solid-phase enzyme immunoassay to detect proteins and target molecules. The ELISA-based sensor has been used as an efficient diagnostic tool in medicine, food safety, analysis of lab, and quality monitoring.

The basic principles of ELISA-based biomarker sensors are as follows: firstly, antigens or antibodies are bound onto the surface of a solid carrier (such as an electrode or substrate) such that their immunologic competence is kept. Then, making an enzyme-labeled antigen or antibody by linking antigen or antibody with an activity enzyme will keep activities of both biomarkers and enzymes. When detecting target biomarkers, the analyte is captured by electrode or substrate by specific binding between antigens and antibodies following certain steps. The antigen-antibody compounds with enzyme on the electrode or substrate are separated from other substances by washing, and the amount of enzyme combined with the solid phase carrier is proportional to the amount of enzyme that is decorated onto target biomarkers. After adding the substances that can react with enzymes specifically, these substances will be catalyzed by enzymes and converted into some colored products. The amount of colored products is directly related to the concentration of target biomarkers, so it can be analyzed qualitatively or quantitatively according to the depth of the color. Because of the high catalytic efficiency of the enzyme, the reaction effect can be greatly enlarged; hence, the ELISA-based biomarker sensors are characterized by high sensitivity.

**Figure 7:**
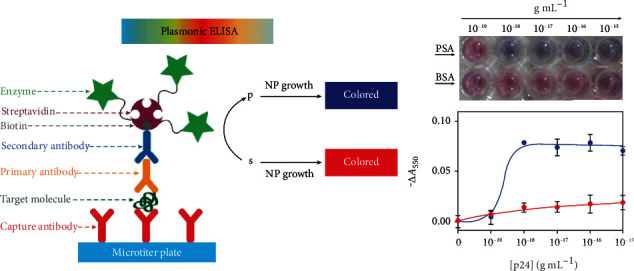
Schematic of ELISA. The biocatalytic cycle of the enzyme generated color nanoparticle solutions of characteristic tonality (S: substrate; P: product; NP: nanoparticle) The figure is reproduced from De La Rica and Stevens [117] with permission.

As shown in Figure 7, De La Rica and Stevens introduced a novel signal generation mechanism for biosensing that enabled the detection of a few molecules of the analyte with the naked eye. In their work, the growth of gold nanoparticles and colored solutions with distinct tonality was controlled by the enzyme label of biomarkers [117]. Combining with modern material, Wu et al. enhanced fluorescence ELISA according to the human alpha-thrombin triggering fluorescence “turn-on” signals. In their work, Ab2 and HAT acted as detection labels which were decorated onto the gold nanoparticles, and a bisamide derivative of Rhodamine110 with fluorescence quenched worked as the substrate of HAT [118]. Liang et al. developed a plasmonic biosensor for PSA detection based on the triangular silver nanoprism (AgNPRs). By measuring the shift of SPR peak, the target analyte could be quantitatively detected according to the changes of the color [119]. Aside for the material-based improvement, the microfabrication and microstructure also provide some new ideas in the ELISA-based biosensor. Arya and Estrela developed an electrochemical ELISA biosensor for NUMA1 and CFHR1 detection with an off-site matrix fluidic structure. During the detection, the interactions occurred in the channel apart from the sensing electrode, thereby reducing the electrochemical background signal [120]. Ghosh et al. developed a microchannel capillary flow assay to detect the malaria biomarker PfHRP2. In their work, the functionalized microchannel not only allowed a sequential flow due to its hydrophilicity of the surface but also eliminated its shortcomings by dealing with the liquid reagent [121]. Sanjay et al. successfully fabricated a microfluidic immunoassay for IgG and hepatitis B surface antigen detection. In their work, the capture efficiency of biomarkers is enhanced due to the microfluidic channel which allows the analyte to pass through the sensitive area multiple times [122]. Compared to traditional ELISA, Yu et al. fabricated a metal-linked immunosorbent assay by replacing the traditional enzyme as the chemical dissolution of Ag nanoparticles to amplify signals. As a kind of enzyme-free ELISA, their work exhibited about two magnitudes higher sensitivity and 4 times faster for chromogenic reaction than traditional ELISA [123]. Cui et al. reported a new method that combined nanoparticle-assisted acoustic molecular concentration with ELISA to realize a concentration factor exceeding two orders magnitude within the 30s for no-wash cancer biomarker detection [124].

The biomarker sensor based on ELISA has ultrahigh sensitivity and ultralow detection limits. At the same time, through research and development in recent years, the ELISA kit has been widely used in the medical field and personal early diagnosis and rapid screening because of its easy commercialization and stable preservation.

## 4. Surface Plasmon Resonance-Based Biomarker Sensor

The principle behind electrochemical biomarker sensors is the electrode charge transfer or energy transfer after the specific binding of biomarkers. However, the mechanism of SPR-based sensors is the change of material properties or electronic states of sensors caused by antigen-antibody specific binding, which produce different electrical signals at different concentrations. Based on well-established microfabrication techniques, various kinds of biomarker detection mechanisms have been developed based on the basic principle of SPR [125–132].

SPR is an optical physical phenomenon; the surface of the metal generates plasma waves when the polarized light reflects with a certain angle onto the metal film. It will produce the metal surface resonance when the propagation coefficient of the incident electromagnetic wave is matched with the metal. When the SPR is applied to the biomarker detection field, the biomarker specific binding will change the refractive index of the plasma resonant material and make a change in the resonant angle of the SPR which can be measured to detect the concentration of biomarker. SPR-based biomarker sensors always have ultrahigh sensitivity and resolution; they are always used in the laboratory and in the precise analysis area, rather than personal early diagnosis.

**Figure 8:**
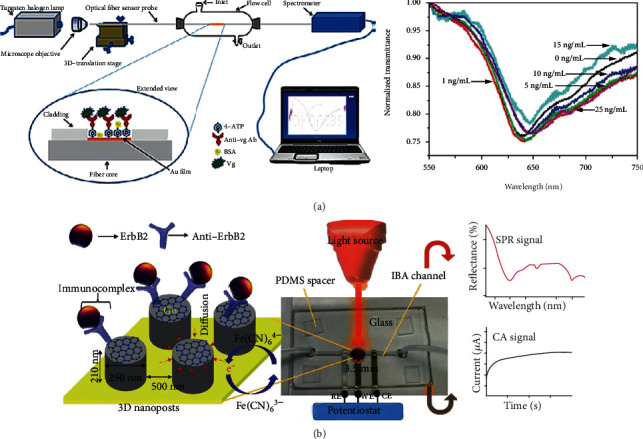
SPR-based biomarker sensor. (a) is a schematic of a fiber-optical SPR-based biomarker sensor and the detection results with different concentration of Vg from Srivastava [133] with permission and (b) GO sheets and 3D nanoposts are fabricated to enhance the SPR and form amide bonds with -NH2 groups of anti-ErbB2, reproduced from Ali et al. [134] with permission.

As Figure 8(a) shows, Srivastava fabricated a fiber-optical SPR biosensor for detection of an endocrine disruption biomarker—vitellogenin—in aquatic environments [133]. Cennamo et al. fabricated a low-cost biosensor for vascular endothelial growth factor (VEGF) detection based on SPR technology with a plastic optical fiber waveguide. In their experiment, two crucial points were proposed to improve the sensor: on the one hand, the functional layer with DNA aptamers could bind the high affinity and specificity nonnucleic acid targets due to its short oligonucleotide sequences; on the other hand, a planar gold layer was fabricated as the sensing area of the POF [135].

Combined with other 2D materials, some SPR-based biomarker sensors made great progress. As is shown in Figure 8(b), taking advantage of graphene oxide, Ali et al. proposed a dual-modality microfluidic sensor for biomarker detection. In their work, the periodic gold nanopost array was assembled with graphene oxide and provided both SPR and electrochemical signals in one platform [134]. He et al. fabricated a SPR biosensor for folic acid protein (FAP) detection. In their work, due to the interaction between folic acid receptors and graphene through *π*-stacking, the novel 2D material—graphene—had been used for the SPR chip to improve the sensitivity and selectivity of FAP in the serum [136]. Taking advantage of graphene's highly efficient light absorption ability, Hossain et al. proposed a simulation model of graphene-coated fiber-optic SPR biosensor for early breast cancer detection. From the variations of the SPR angle and surface resonance frequency, this biosensor shows excellent selectivity between the probe DNA and the target DNA [137].

Taking advantage of microfabrication technologies, Liu et al. enhanced near infrared fluorescence 50-fold with a plasmonic gold chip compared to traditional methods. Lung cancer biomarkers were detected from 10 *μ*L of human serum on plasmonic gold chip and achieved marked improvement on limit of quantification, limit of detection, reproducibility, and sensitivity [138]. Li et al. improved the localized surface plasmon resonance (LSPR) properties of the sensor based on the nanoporous structure and subwavelength nanoparticle. In their work, a novel nanoporous gold pattern was proposed by using localized laser heating, which enabled the ability to pattern nanoporous gold with various sizes and shapes by measuring parameters such as laser power, irradiation duration, and solution environment [139]. Based on the microcontact imprinting (MIP), Ertürk et al. fabricated a novel surface plasmon resonance biosensor for PSA detection with ultrahigh sensitivity [68].

In recent years, combining microfabrication technology and materials science, the SPR-based biomarker sensor continues to break the bottleneck and improve its performance. With the minimization of SPR-based biomarker sensors, some commercial SPR-based sensors will be developed and even as extremely accurate detection devices are used in the field of personal physiotherapy and early rapid cancer detection. Although nowadays SPR-based biomarker sensors are limited by their poor anti-interference ability, they still show great prospects in biology information detection.

## 5. Microstructure-Based Biomarker Sensors

With the advent of artificial intelligence, smart microchips and smart application devices have become a powerful driving force to promote scientific development in recent years. The fabrication of these microchips and sensors obviously depend on the continuous development and innovation of microfabrication technologies. Based on standard microfabrication technologies (such as lithography, deposition, growth, etching, and stripping), many more microstructure-based sensors are used in the field of biomarker detection, such as cantilever beam, nanopore, interdigitated electrode, and field-effect transistor, whereas some micro- or nanostructure-based on nonstandard microfabrication technologies even expand the design space of biomarker sensors. Besides that, combined with the development of two-dimensional materials in microelectronics, more microstructure-based sensors have been developed, such as nanowire field-effect transistors.

The research of different theories also provides a platform for microstructure-based sensors, for example, combining with hydrodynamics theory, some microfluidic or nanofluidic biosensors have been developed, and some more metadevices with micro- or nanolevel based on the theory of electromagnetic metamaterials which work in the range of ultrahigh frequency and have been applied in various fields including biomarkers detection. These minimization biomarker sensors always exhibit different physical features compared to traditional devices and show excellent performance in biomarker detection.

### 5.1. Field-Effect Transistor-Based Biomarker Sensors

Field-effect transistor (FET) is one of the classic microstructure sensors based on microfabrication technologies, which is widely used in integrated circuits and microchip fabrication. Nanowire-based FET structure is the most closely related to biomarker detection that has been reported in many research studies.

Nanowire-based sensors are always fabricated by standard microfabrication technologies, such as growth and etching. Due to large surface-to-volume, nanowire-based FETs can capture more biomarkers per unit volume, thus enhancing their sensitivity for target detection. Furthermore, combined with the characteristics of ultrafast electron transport of two-dimensional materials such as graphene, nanowire-based FET biomarker sensors have obvious advantages in LoD and response time compared to traditional biomarker sensors.

Generally, the target biomarker is immobilized onto the surface of the nanowires, and free charges will be transferred between biomarkers and nanowire and exhibit different I-V characteristics under certain bias voltages in FET.

**Figure 9:**
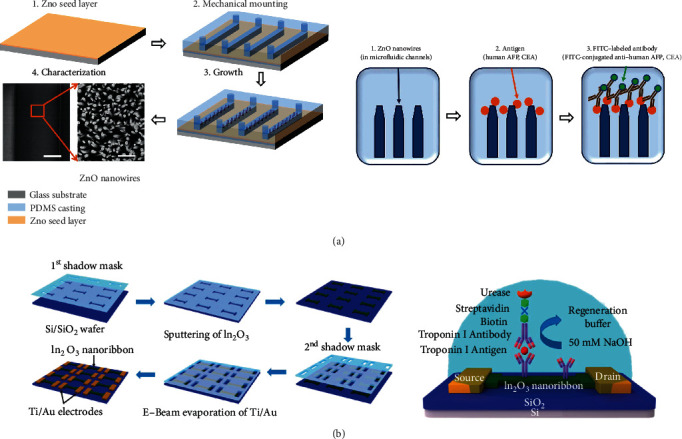
Nanowire-based FET biomarker sensor. (a) is a schematic of the ZnO nanowire-based biosensor which was combined with microfluidic technology, reproduced from Guo et al. [140], and (b) is the FET-like biosensor which is fabricated by sputtering and E-beam evaporation with multiple shadow masks, reproduced from Liu et al. [141] with permission.

Gao et al. developed a multiplexed lung cancer biomarker sensor based on the standard nanowire FETs, which was fabricated by complementary metal-oxide-semiconductor (CMOS) technologies. In addition to nanowires, more and more self-assembled biomarker sensors based on the classical FET structure have been developed [142]. Lin et al. developed an ultrasensitive biomarker sensor based on polypyrrole, a flexible conductive network decorated with polymeric composites to detect a melanoma cancer biomarker—CRP. As a signal amplifier, the polypyrrole nanowire based sensor could detect serum C-reactive protein (CRP) levels through monitoring the conductance change [143]. As is shown in Figure 9(a), with a larger binding surface area and intrinsic fluorescence enhancement, Guo et al. developed a ZnO nanowire FET biosensor to detect both AFP and CEA with varying diameters and lengths of ZnO, in which the ZnO nanowire was successfully fabricated by controlling the content of polyethyleneimine and growth time in microfluidic channels [140]. Through the detailed research on surface functionalization and covalent immobilization of biomarkers, Janissen et al. fabricated an InP nanowire-based FET biosensor. By using biocompatible ethanolamine and poly(ethylene glycol) derivate coatings, both the sensitivity and molecular selectivity were substantially increased. Due to the low surface recombination rates of InP nanowires, it could have been used as the new active transducer for many biosensors, and the LoD of Chagas Disease protein marker (IBMP8-1) had been dramatically optimized [144]. Lu et al. proposed a high sensitivity silicon nanowire (SiNW) FET array for CYFRA21-1 and PSA detection, in which the double-channel PDMS microfluidic and SiNW are all compatible with CMOS technologies [145]. In order to improve electrical performance and increase the sensing area, a honeycomb-like nanowire structure was fabricated by Kim et al. to detect cardiac troponin I, which exhibited high On-Off current ratio, small subthreshold swing, and low gate leakage current [146]. Zhang et al. developed a multiplexed biomarker sensor for IL-8 and THF-a detection by using SiNW FET, and the LoDs of both proteins were 10 fg mL^−1^ in the buffer solution and 100 fg mL^−1^ in artificial saliva [147]. An ultraflexible stretchable field-effect transistor nanosensor is presented by Wang et al., who used aptamer-functionalized monolayer graphene as the conducting channel. Since the specific binding could cause change of the graphene's carrier concentration, the biomarker concentration can be successfully determined [148].

### 5.2. Metamaterial-Based Biomarker Sensors

The metamaterial is a “material” that is artificially engineered to have a property that is not found in nature [15], which is usually assembled by multiple periodical metal arrays with microfabrication [149]. Due to these metallic units that are much smaller than the wavelength of incident electromagnetic waves, the periodic array structure is considered as the homogeneous “material.” Therefore, the characteristics of metamaterials can be represented by equivalent permeability and equivalent conductivity. As a result, the metamaterial is always the “double negative” material; it can interestingly influence electromagnetic wave and display striking electromagnetic properties like backward propagation, reverse Doppler effect, reverse Vavilov-Cherenkov effect, negative refraction, diffraction-limit breaking imaging, invisible cloak [150–154], and so on. In general, metamaterial-based sensors provide a new method for biosensing systems and promote the progress of biosensor detection sensitivity to single molecule detection. Through its high-throughput array structure, sensors based on electromagnetic metamaterials not only bring new modalities for biological imaging but also provide technical support for other detection technologies. From the perspective of medical diagnosis, the detection of a single biomolecule will completely change the diagnosis and treatment of various diseases [16].

By researching electromagnetic metasensors, it is necessary to study how to design a pattern or structure to optimize transport properties. In the split-ring resonator- (SRR-) based biomarker detection, the transmission forbidden band is the focus of the research. Researchers try their best to achieve a better quality factor (*Q* factor) to improve the detection accuracy and sensitivity. Analyzing the principle of SRR-based biomarker sensor from another perspective, the specific binding between antigen and antibody provides the surface of the metallic structure with extra charges which impact the I-V transmission curve, and more importantly, due to the incomplete matching of the dielectric constants between the antigen and the antibody, these biomarkers bring additional parallel or series capacitance to the metal structure that seriously affects the magnetic resonance frequency. Therefore, the larger the concentration, the greater the resonance frequency shifts.

Based on the basic principle of SRR metamaterials, some researchers tried to make some detailed discussions about mechanisms and prospects by simulation. Saxena and Daya built a theoretical model of concentric square ring with the biological material and made the simulation to study the property of this biological material through computer simulation technology microwave studio [158]. Wellenzohn and Brandl built a simulation model by using the finite element method and designed a novel structure-Cu/FR4/Cu/Ni/Au to detect permittivity changes in the bio-layer [159].

**Figure 10:**
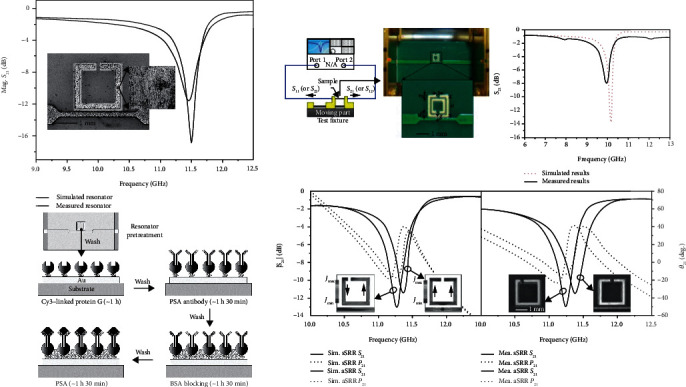
Metamaterial-based (SRR) biomarker sensor. These sensors were all based on SRR and its derived structures, and the concentrations of biomarkers can be obtained by measuring the drifts of resonance frequency. Reproduced from Lee et al. [155–157] with permissions.

Based on theoretical work, some biosensors with metamaterials had been gradually developed in recent years. As Figure 10 shows, Lee et al. exhibited various SRR-based biomarker sensors with different patterns, such as symmetric single ring, double ring and asymmetric single ring. In their works, the frequency shifts were obtained at different concentrations of biomarkers and calculated to show excellent results for the detection of PSA, cortisol, and alpha-amylase, respectively [155–157]. Due to the tunable plasmonic metamaterial provided double-channel for both optical and SERS transmissions in Cao et al.'s work, the sensor could monitor conformational states and binding affinity of biomolecules simultaneously. And then, they demonstrated this sensor also can be used in fingerprint analysis and biomarker detection in different environments [160]. Combing with a PDMS microchannel, Jaruwongrungsee et al. fabricated a real-time and label-free IgG sensor based on the split-ring resonator coupled with a microstrip structure [161]. Torun et al. fabricated a metamaterial biosensor based on split-ring resonator coupled with an antenna. In this microwave biosensor, a single circular SRR had been used as the sensing part and monopole antenna on the both side of the substrate acted as the transmitter and receiver [162]. Harnsoongnoen and Wanthong fabricated a biosensor for D-glucose detection based on a metamaterial pattern, which worked as an electric LC resonator. In their work, the detection results showed wide dynamic range and high linear relationship between the concentration of D-glucose and shift of resonance frequency [163]. Taking advantage of print circuit board (PCB) fabrication technique, Camli et al. fabricated a split-ring resonator on the FR-4 substrate for the detection of glucose. The resonant frequency's redshift of the sensor occurred after the combination of glucose oxidase enzyme and glucose [164]. In Ekinci et al.'s work, a flexible substrate had been used to study the effect of curved surface on resonance frequency of the SRR; they successfully fabricated a strain sensor for glaucoma detection. Their work proved that the flexible metamaterial sensor can also be applied into the conventional lens field [150].

As microfabrication developed, some researchers fabricated metasensors in the size of the nanometer and the resonance frequency was increased to the THz range. Because of its excellent detection specificity and non-destructive effect on biomolecules, terahertz electromagnetic waves have obvious advantages over traditional microwave sensors in biomarker detection. Ahmadivand et al. developed a THz metasensor for detection of trace target biomarker based on a plasmonic surface with metamaterial units. In their work, GNPs were decorated with the same antibodies onto the units of metamaterials to enhance the capturing of biomarker molecules, and 100-fold sensitivity enhancement was obtained in comparison to analogous devices [165]. To reduce the absorption of water to terahertz electromagnetic wave, Geng et al. introduced the microfluidics technology into their THz metamaterial-based biosensor and successfully improved the detection sensitivity [166]. Hu et al. fabricated a metamaterial resonator to monitor the interaction between BSA solution and four different kinds of drug solutions in real-time with terahertz time-domain spectroscopy system [167].

With the continuous deduction of metamaterial theory, more and more design methods will be proposed, and applications based on these methods will play a crucial part in the field of biological detection. In the biosensor field, the background noise (especially the solution environment) is the main problem that limits the application of metasensors in actual detection.

### 5.3. Nanopore Biomarker Sensors

Nanopore is one of the classical nanostructures developed for many years. Based on its electrical characteristics, when the biomarker passes through the nanopore, the nanopore sensor can easily detect the change of the current. After measuring these output signals, the target biomarker can be detected and even achieve single molecule detection [168].

Ali fabricated a bare nanopore biosensor for detection of epidermal growth factor receptor (EGFR). In the work, the anti-EGFR aptamer was used to optimize its selectivity caused the reduction of dwell time by 23% [169]. Combining with molecular dynamics, simulations and special algorithms of signal processing, Sarathy et al. proposed a novel method to classify three different epigenetic biomarkers based on nanopore. In their work, the double-stranded DNA was used as a matched filter to improve its selectivity of these target biomarkers [170]. Lin et al. fabricated a nanopore biomarker sensor for the detection of PSA without any pretreatments. The high selectivity of the DNA probe and nanoporous membrane was used to improve the selectivity and generate a deeper current blockade [171]. Combining with the modified clustering algorithm and hidden Markov model, Zhang et al. successfully developed a nanopore biosensor for multilevel current blockage measuring. Based on this strategy, the accuracy of the biosensor had been improved and showed a promising performance to identify the multiplex current blockages [172]. Yu and coworkers fabricated a biomarker sensor based on a quartz nanopipette for protein detection. In their work, AFP, as the target biomarker, was detected with or without its antibody in a label-free method [173].

In addition to the artificial nanopore, some biological nanopores can be used to detect some biomolecules. Huang and his coworkers detected some protein biomarkers which are from 25 kDa to 1.2 kDa based on Fragaceatoxin nanopores driven by electroosmotic flow [174]. Liu et al. fabricated a DNA-assisted nanopore sensor for multiplex biomarker simultaneous detection. In their work, some biomarkers such as PSA, CEA, and AFP linked with different barcodes of DNA to exhibit current blockages [175].

As a classic MEMS sensor, nanopore has the advantages of fast fabrication and simple testing. Whether these nanopore biosensors are based on silicon and its derivative materials, biological materials, or some porous materials, the structure themselves have ultrahigh sensitivity to ion transfer, so that the nanopore sensor plays an important role in biological detection, such as DNA, mRNA sequencing, and biological macromolecule recognition. In recent years, combining with various fabrication technologies and nanoporous materials, some researchers have made major progress in reducing response time and increasing output signals. Meanwhile, combined with some special algorithms, the data processing capabilities of nanopore biomarker sensors were improved, which improves the accuracy in the detection of biological macromolecules.

However, existing nanopore biomarker sensors still have some problems. As the electrochemical biomarker sensor, the operation conditions of the nanopore biomarker sensor would destroy the activity of some of the biomarkers to a certain extent, resulting in the deviation of real concentration. Since the nanopore biomarker sensor is dependent on its pore size, it is hard to control by an active method, which limited its applications to multiplex biomarker simultaneous detection. The response time and translocation of biomarkers have always been a contradiction that cannot be ignored. Researchers hope to shorten the response time to make the high-throughput detection and want to reduce the translocation of the biomarker to obtain more accurate output signals. Finally, in order to improve the selectivity of nanopore biomarker sensors, pretreatment of nanopores or biomarkers is required, which also limits its applications in biomarker detection.

## 6. Other Biomarker Sensors

Besides the biomarker sensors mentioned above, there are many other biomarker sensors with interesting designs and ideas. Sinibaldi et al. successfully demonstrated and achieved label-free detection of the cancer biomarker Ang2 based on Bloch surface waves on functionalized 1D photonic crystals biochips, which take advantage of specific geometry to obtain maximum figure of merit (FOM) and minimum LoD [176]. Liu et al. developed a label-free fiber-optic biosensor to detect cTn-1 by using the phase-shifted Bragg grating in microfiber. Besides the physicochemical method, some computer engineering arithmetic had also been used to optimize the performances of the biomarker sensor in their work [177]. Sun and coworkers developed a rapid, label-free, and high sensitive biomarker sensor for HER2 detection both in phosphate-buffered saline (PBS) and serum by using silica microfiber interferometry [178].

Washburn et al. realized the function of biomarker detection by a silicon photon sensor and obtained the concentration of biomarker by measuring the refractive index of the sensor surface [179]. Diware et al. developed a solution-immersed silicon biosensor for myocardial infarction early diagnosis. Due to this sensor having a very high sensitivity on the thickness of the biolayer under the nonreflection condition, it would become a candidate for detecting biomarkers or other biological information from humans [180].

Wang et al. demonstrated a label-free ErbB2 sensor, which combined both biomolecule detection and microchannel by using a SOI-based metasurface. The different concentrations of biomarkers caused different wavelengths of the sensor and the reflectance change as the changes of the incident angles [181]. Liang et al. came up with an ultrasensitive detection method for pancreatic cancer biomarkers (CA 19-9, CA 242, proANP, and BNP) by multiphoton nonlinear laser wave-mixing spectroscopy technology with an excellent signal-to-noise ratio and is label-free [182].

As a proven effective method, surface-enhanced Raman spectroscopy (SERS) technology has also been successfully used in the biomarker detection field. By using two machine learning algorithms (*K*-nearest neighbor and classification tree), Banaei et al. optimized the data processing of the protein biomarker detection platform and improved the repeatability and selectivity from analysis results based on SERS liquid biopsy assay [183]. Nirala and Shtenberg developed a quantitative detector of CEA based on the SERS technology. To enhance the electromagnetic characteristic of SERS, the two-layer nanoparticles with core and shell structure were decorated onto the substrate of the sensor which generated a large amount of “hot spots” in their work [184]. Ganesan et al. successfully created a unique biocompatible aluminum-based quantum structure for cancer cell detection based on SERS. Due to the wrinkled Al QDs was established to be the best for SERS activity, it could even achieve single-molecule detection in their work [185].

Due to the high enhancement factor of rationally designed plasmonic nanostructures, a single detectable nanotag had been used in the work of Zhang et al. And then they fabricated a biosensor for a multiplex of cardiac biomarkers detection with core-shell SERS nanotags which could amplify the signal and was much more stable [186]. Chang et al. demonstrated a sensitive immunoassay using SERS active nanoparticles which were sufficiently sensitive and photostable to be detected at the single probe level [187]. Combining SERS technology with surface molecularly imprinted polymer (MIP) technology, a novel biosensor was fabricated for CEA detection by Lin et al. [188]. Taking advantage of surface molecularly imprinted polymer, recognition sites with high affinity to the analyte enhanced stable and specific capture ability. Base on the MIP method, Ertürk et al. developed a capacitive immunosensor for real-time bioinformation monitoring [189].

Combing with QDs and NPs, some novel biosensors have been designed and fabricated. Ryu et al. developed a dual emission fluorescent sensor for Bacillus anthracis spore detection. In their work, a macromolecule complex-EuIII was decorated on the gold QDs to increase the surface to volume ration; more than that, the ultrafine particles (Eu-GQDs) also enhanced dispersibility and improved detection sensitivity [15]. Zhao et al. modified the biomarkers with gold nanoparticles (GNPs) to locate and detect biomarkers by scanning the frequency shift and intensity of UV spectroscopy [190]. Takano et al. demonstrated a high-sensitivity PSA sensing platform that protein A was immobilized on the reaction plate for enhancing efficient immobilization of anti-PSA and three different fluorescence-secondary labels also were used to optimize the performances of this platform [191]. In addition, the nanoparticle assisted lateral flow technology used in the biomarker detection field. Chen et al. proposed a chemiluminescent-gold lateral flow sensor for AFP and CEA detection with a novel signal amplification strategy. In their work, GNP was used and decorated with HRP and Ab1 to improve the traditional lateral flow based sensor with the increase of sensitivity by three orders of magnitude [192]. Yang and coworkers fabricated a paper-based barcode assay for detection of multiplex target biomarker combing with GNP, and this sensor also showed its potential in the point-of-care field with the low-cost, rapid detection, and good stability [193].

## 7. Summary and Conclusions

Human health is one of the most important research topics. As one of the most effective methods, biomarker detection has important significance in the early diagnosis of disease and individual health monitoring. Many biomarker sensors have been developed in recent years, such as electrochemistry-based sensors, SPR-based sensors, and metamaterial-based sensors.

The chemical biomarker sensors require enrichment and extraction of analysts before detection and analysis. In a detection platform, biomarkers can be directly observed by electron microscopy through some preprocessing (such as fluorescent labeling). Therefore, chemical biomarker sensors always have high accuracy. However, the time-consuming read-out process and strict detection environment limit individual applications.

Electrochemical biomarker sensors are widely used in many areas due to their low cost and high accuracy, which could provide individuals with early diagnosis in a convenient environment. Although combined with various nanomaterials and MEMS technologies, several novel biomarker sensors have been developed with ultrahigh LoD and excellent linear ranges; there also exist some problems for practical application. Firstly, some nanomaterials are limited by pH, temperature, etc., which lead to poor stability and worse tolerance of temperature and pH conditions. Secondly, the two-dimensional composite materials have poor stability for long storage, which decreases their stability and also limits the commercialization of such sensors. Thirdly, some electrodes create many wrinkles and defects in nanomaterials, which greatly reduce the electrical conductivity in turn. Moreover, the aqueous environment may damage nanomaterials, which leads to biosensors' error or disability. Finally, some of NPs or 2D materials in the composite material inhibit or interact with the biomarker, which affects the activity of biomarker or specific binding, resulting in a discrepancy of actual concentration [28, 34, 35, 39, 119, 187, 190].

Combined with microfabrication technologies, the microelectromechanical system (MEMS) also demonstrates great potential in biomarker detection. As shown in Table 3, these MEMS-based biosensors exhibit ultrahigh sensitivity and excellent LoD because of the high sensitivity to electric transfer. However, comparing with Si-based MEMS fabrication, these novel nanomaterials and nonstandard fabrications have poor repeatability, which leads to the instability and poor repeatability for their application in the real detection environment.

Among these MEMS biomarker sensors, metamaterial-based biomarker sensors show great potential, which can achieve not only ultrahigh precision detection but also personal real-time detection by reducing the cost of standard microfabrication technology. In recent years, terahertz biosensors based on metamaterials have been extensively developed. These sensors which work in the terahertz can minimize damage to biomarkers and greatly promote the development of noninvasive detection.

To summarize, combined with the optimized labeling technology, research of new materials, and advanced MEMS technology, some novel biomarker sensors had been investigated in recent years. Although they exhibit the excellent performance of LoD, sensitivity, and linear range, there is still a long way to go before they can be used for early diagnosis of cancer and individual health monitoring.

## 8. Future Perspectives

Although a large number of biomarker sensors have been developed in recent years, the performance of commercial sensors applied in the clinical field has not improved significantly. Considering modern medical diagnosis requirements, which are mentioned in Introduction, they require us to pay more attention to the development and optimization on the practical commercial sensor compared to the laboratory products. As the incidence of various cancers continues to change, single-biomarker detection is clearly no longer sufficient for diagnostic requirements. Therefore, fast, multilabel, and simultaneously accurate detection is the most efficient way to connect laboratory products with clinical medicine.

In the following research, we can try to combine micro- or nanofluidic technology to increase device stability and try to improve the design of SPR miniaturization to achieve an accurate real-time on-site detection and also try to vigorously develop terahertz biomarker sensors based on metamaterials to realize “body health scanning” with a noninvasive detection method. We have the confidence to believe that, as the development of materials science and microfabrication technologies continues, some practical biomarker sensors could break through the bottlenecks, optimize performance, and achieve breakthroughs for human health in the future.

## Figures and Tables

**Table 1:** Concentration of common biomarkers in humans.

Biomarker	Concentration in humans	References
Prostate-specific antigen (PSA)	Reference range: 0.5-2 ng/mL	[8–10]
Interleukin 6 (IL-6)	Reference range: <6 pg/mL	[11]
Carcinoembryonic antigen (CEA)	Reference range: 3-5 ng/mL	[12]
*α*-Fetoprotein (AFP)	Reference range: 0-20 ng/mL	[13]

**Table 2:** Electrochemistry-based sensors for PSA, CEA, and AFP detection.

Target	Strategy	LoD	Linear range	References
PSA	EM	2 pg/mL	3 pg/mL-60 ng/mL	[28]
	EM	1 pg/mL	100 pg/mL-100 ng/mL	[40]
	EM	—	0.1 pg/mL-0.1 mg/mL	[64]
	EM	0.29 pg/mL	1 pg/mL-10 ng/mL	[30]
	EM	0.03 pg/mL	0.1 pg/mL-10 ng/mL	[49]
	EM	0.03 pg/mL	0.1 pg/mL-100 ng/mL	[50]
	EM	0.25 fg/mL@SWSV 3.04 fg/mL@DPSV	—	[48]
	BD	15 fg/mL	—	[99]
	DS	100 fg/mL	—	[105]
	DS	0.31 pg/mL	0.001 ng/mL−100 ng/mL	[115]
	DS	16.6 fg/mL	50 fg/mL-40 ng/mL	[113]
	DS	1 ng/mL	1 ng/mL-100 ng/mL	[114]
	DS	2.6 pg/mL	5 pg/mL-50 ng/mL	[116]
	DS	17 fg/mL	0.05 pg/mL-100 pg/mL	[112]

CEA	EM	0.5 pg/mL	0.025 ng/mL–25 ng/mL	[42]
	EM	0.23 ng/mL	1.0 ng/mL-25.0 ng/mL	[35]
	EM	8 pg/mL	12 pg/mL-85 ng/mL	[28]
	EM	—	0.05 pg/mL-1.25 pg/mL	[70]
	EM	1.4 pg/mL	0.005 ng/mL-20 ng/mL	[43]
	BD	1.8 pg/mL	—	[93]
	BD	1.90 fg/mL	2 fg/mL-500 fg/mL	[100]

AFP	EM	3 pg/mL	0.01 ng/mL-100 ng/mL	[26]
	EM	0.096 ng/mL	0.8 ng/mL-10 *μ*g/mL	[69]
	BD	0.3 pg/mL	—	[93]
	BD	1.36 fg/mL	2 fg/mL-500 fg/mL	[100]
	BD	122.4 fg/mL	136 fg/mL-34 pg/mL	[96]
	BD	0.2 pg/mL	0.4 pg/mL-40 ng/mL	[98]
	DS	0.33 pg/mL	1 pg/mL-20 ng/mL	[103]
	DS	0.01 pg/mL	0.02 pg/mL-10 ng/mL	[108]
	DS	6.7 fg/mL	20 fg/mL-100 ng/mL	[110]

EM: electrode modification; BD: biomarker decoration; DS: double strategies.

**Table 3:** Other sensors for PSA, CEA, and AFP detection.

Target	Strategy	LoD	References
PSA	SPR	91 pg/mL	[68]
	ELISA	0.001 fg/mL	[117]
	ELISA	4.1 fg/mL	[119]
	ELISA	0.1 ng/mL	[123]
	Other (SERS)	0.11 pg/mL	[187]
	Other (fluorescence)	10 pg/mL	[191]
	Other (MIP)	0.08 pg/mL	[189]
	FET	1 fg/mL	[145]
	Nanopore	25 fg/mL	[171]
	Nanopore	37 fg/mL	[175]

CEA	FET	100 fg/mL	[140]
	MMs	1 ng/mL	[155]
	MMs	100 pg/mL	[156]
	Other (lateral flow)	0.17 ng/mL	[192]
	Nanopore	1.25 pg/mL	[175]

AFP	ELISA	0.01 fg/mL	[118]
	ELISA	0.1 ng/mL	[123]
	FET	1 fg/mL	[142]
	FET	1 pg/mL	[140]
	Other (SERS)	0.064 pg/mL	[184]
	Other (lateral flow)	0.21 ng/mL	[192]
	Nanopore	136 fg/mL	[175]

## Abbreviations

Ab1: Primary antibody
Ab2: Secondary antibody
AFP: *α*-Fetoprotein
AgNPR: Silver nanoprism
ALP: Alkaline phosphatase
BSA: Bovine serum albumin
CCD: Charge-coupled devices
CEA: Carcinoembryonic antigen
CMOS: Complementary metal oxide semiconductor
CNTs: Carbon nanotubes
CRP: C-Reactive protein
CSs: Carbon spheres
CVD: Chemical vapor deposition
ECL: Electrochemiluminescence
EGFR: Epidermal growth factor receptor
EIS: Electrochemical impedance spectroscopy
ELISA: Enzyme-linked immunosorbent assay
ErbB2: Erythroblastic leukemia viral oncogene homolog 2
FAP: Folic acid protein
FET: Field-effect transistor
FOM: Figure of merit
g-C_3_N_4_: Graphitic carbon nitride
GCE: Glassy carbon electrode
GNPs: Golden nanoparticles
GO: Graphene oxide
HCR: Hybridization chain reaction
HER2: C-ErbB2
HRP: Horseradish peroxidase
IBMP8-1: Chagas disease protein
IgG: Immunoglobulin G
IL-6: Interleukin 6
IL-8: Interleukin 8
ITO: Indium tin oxide
LoD: Limit of detection
LSPR: Localized surface plasmon resonance
MEMS: Microelectromechanical system
MIP: Molecularly imprinted polymer
MMs: Metamaterials
MWCNTs: Multiwalled carbon nanotubes
NDPK-A: Nucleoside diphosphatase kinase A
NPs: Nanoparticles
NWs: Nanowires
PBS: Phosphate buffered saline
PCB: Print circuit board
PDMS: Polydimethylsiloxane
PEC: Photoelectrochemistry
PMMA: Polymethyl methacrylate
PSA: Prostate-specific antigen
PSMA: Prostate-specific membrane antigen
TNF-*α*: Tumor necrosis factor-alpha
*Q* factor: Quality factor
QDs: Quantum dots
rGO: Reduced graphene oxide
SERS: Surface-enhanced Raman scattering
SiNW: Silicon nanowire
SOI: Silicon-on-insulator
SPR: Surface plasmon resonance
SRR: Split ring resonator
VEGF: Vascular endothelial growth factor.
